# Prevalence of Three-Rooted Mandibular First Molars Among Jordanian Royal Medical Services Patients Using Cone-Beam Computed Tomography

**DOI:** 10.7759/cureus.73747

**Published:** 2024-11-15

**Authors:** Khuzama Abu Rumman, Nesreen Al Faraieh, Ghufran A Al-Bataineh, Anas I Abu Salem, Yazan B Shdefat, Wael A Alawneh, Saif Aburumman

**Affiliations:** 1 Endodontics, King Hussein Military Hospital, Jordanian Royal Medical Services, Amman, JOR; 2 Endodontics, Jordanian Royal Medical Services, Amman, JOR; 3 Periodontology, Jordanian Royal Medical Services, Amman, JOR; 4 Endodontics, Private Clinic, Amman, JOR; 5 School of Medicine, The University of Jordan, Amman, JOR

**Keywords:** anatomical variation, jordan, mandibular molars, radix entomolaris, radix paramolaris

## Abstract

Background

Anatomical variations in the mandibular first molars can significantly impact endodontic outcomes. The presence of additional roots, such as the distolingual radix entomolaris (RE) and the mesiobuccal radix paramolaris (RP), complicates endodontic procedures. Traditional radiographs often fail to detect these variations due to their inherent limitations. This study aims to determine the incidence of RE and RP in mandibular first molars using cone-beam computed tomography (CBCT) in a Jordanian population.

Methodology

A retrospective study was conducted at the Queen Alia Military Hospital, Jordan, involving 1,278 patients who underwent CBCT imaging from January 2019 to January 2023. We included patients aged 18-70 years with bilateral mandibular first molars. A total of 500 CBCT scans were evaluated by two experienced endodontists to assess the presence of a supernumerary third root. Demographic data, including age, sex, and the presence of right-sided, left-sided, or bilateral third roots, were collected and statistically analyzed using the chi-square and Fisher’s exact tests.

Results

The prevalence of a third root was 7.4% on the right side, 3.2% on the left side, and 2.6% bilaterally. A significant association was found between gender and the presence of a right-sided third root (p = 0.027), with a higher prevalence in females (9.9%) compared to males (4.7%).

Conclusions

The prevalence of this racial anatomical variant was higher in females in the Jordanian population. Clinicians must be aware of this variant. A thorough radiographic examination must be conducted before initiating endodontic procedures to avoid any potential complications.

## Introduction

The primary objective of root canal treatment is to maintain or regain the health of the periapical tissues. The outcomes of root canal treatment are significantly affected in the presence of untreated root/canal [1]. Hence, information regarding the anatomical morphology of teeth ensures the best possible outcome of root canal treatment. The mandibular first molar is the first permanent tooth to erupt and is considered the most frequently involved tooth in endodontic procedures. It displays a considerable amount of anatomic variation regarding the number of roots and root canals [2].

The presence of an additional third root located distolingually in lower molars was first reported by Carabelli in 1844 and was termed “radix entomolaris,” and if an additional root is present mesiobuccally, it was termed “radix paramolaris” [3]. The incidence of radix paramolaris and radix entomolaris has been studied in many studies using extracted teeth or periapical radiographs. Recent studies have investigated the incidence of radix paramolaris and radix entomolaris using cone-beam computed tomography (CBCT) scans [4]. Using CBCT appears to be more accurate and conservative for investigating the prevalence of additional roots in molars. The morphology has been examined across diverse populations and provides valuable clinical insights that aid in improving treatment outcomes [5]. However, dental professionals struggle with accurately diagnosing these anatomical structures due to a lack of knowledge and the limitations of images taken at suboptimal angles [6].

Additionally, previous research has shown a substantial difference in the prevalence of radix entomolaris and radix paramolaris between males and females [7]. Therefore, this study aimed to determine the incidence of radix entomolaris and radix paramolaris in lower first molars and investigate their pattern of concurrence in contralateral molars using three-dimensional CBCT scans in a cohort of Jordanian patients treated at the Jordanian Royal Medical Services.

## Materials and methods

Study design

This retrospective cross-sectional study was conducted in the Department of Dental and Maxillofacial Surgery at the Queen Alia Military Hospital, part of the Jordanian Royal Medical Services in Amman, Jordan. Ethical approval was obtained from the hospital’s institutional review board (IRB), and the study adhered to the Declaration of Helsinki guidelines (IRB approval number: 24-4/2024). Informed consent was obtained from all participants, or their legal guardians if they were under 18 years old, before their CBCT imaging. CBCT scans taken of patients from January 2019 to January 2023 were screened for the prevalence of a supernumerary third root.

Cone-beam computed tomography imaging

The CBCT images were obtained using the Kodak Carestream CS9000® device under standardized conditions, ensuring high-resolution imaging for accurate detection of root structures. Images were obtained in three planes (axial, sagittal, and coronal views) for optimal evaluation of the presence of additional roots. Scans followed the manufacturer’s instructions, with a voxel size of 0.2 mm, a field of view of 5 × 5 cm, and exposure settings suitable for high-resolution imaging of dental structures.

Data collection and evaluation

Demographic data (age, sex) and clinical information regarding the presence of a third root in mandibular first molars were collected. Two experienced endodontists, trained in using the CBCT software, reviewed each image independently. They were blinded to patient information to minimize bias. In cases where discrepancies occurred, a third endodontist was consulted to reach a consensus. The third root was classified as either radix entomolaris (distolingual location) or radix paramolaris (mesiobuccal location), and its presence was recorded for both the right and left mandibular first molars. Additionally, bilateral presence was documented if the third root appeared on both sides. Our population size was 1,278, a sample size calculation revealed a need of 398 patients, our study consisted of 500 patients.

Inclusion and exclusion criteria

The study population consisted of 1,278 patients who underwent CBCT imaging as part of their routine dental care or other diagnostic requirements. Of these, 500 patients met the inclusion criteria. Patients were included if they met the following criteria: (1) bilateral presence of fully developed mandibular first molars, (2) age between 18 and 70 years to ensure complete root formation, and (3) Jordanian nationality to account for demographic consistency. Patients were excluded if they had (1) a history of endodontic treatment or significant restorations in mandibular first molars, (2) missing or unerupted mandibular first molars, (3) incomplete or poorly developed roots, or (4) pathological conditions affecting the mandibular first molars (e.g., resorption, severe caries).

Statistical analysis

All data were analyzed using R statistical software (version 4.4.1). Descriptive statistics, including median and interquartile range (IQR) for continuous variables (e.g., age), were calculated. Categorical data, such as the presence of third roots, their types, and genders, were summarized as frequencies and percentages. Chi-square tests were employed to evaluate associations between categorical variables (e.g., the presence of third roots and sex). A p-value of less than 0.05 was considered statistically significant. Continuous variables were analyzed using t-tests or Mann-Whitney U tests depending on data distribution. Interobserver reliability was calculated using Cohen’s kappa to ensure consistency between evaluators.

## Results

Baseline characteristics

**Figure 1:**
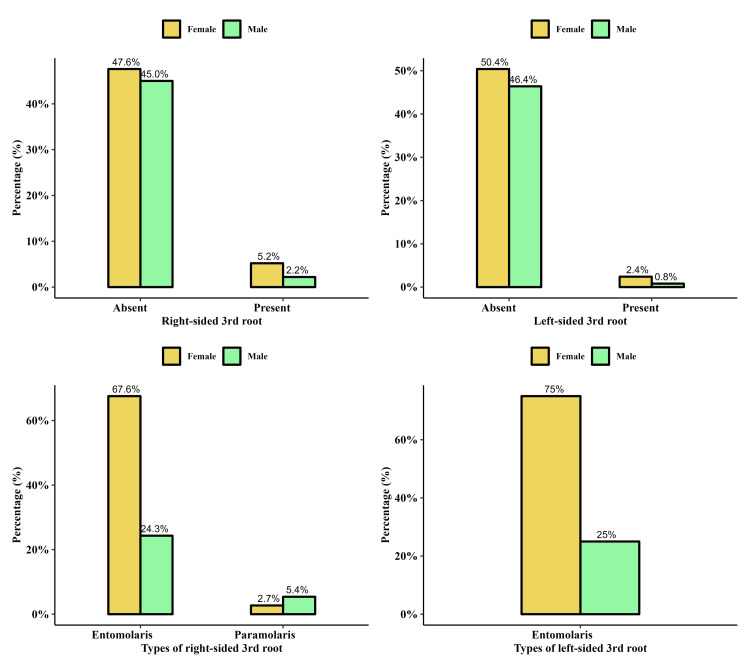
Bar charts showing the distribution of study variables across gender. (A) Right-sided third roots between males and females. (B) Left-sided third root between males and females. (C) Type of right-sided third root between males and females. (D) Type of left-sided third root between males and females.

In the present study, 1,278 CBCT images of Jordanian citizens were analyzed, with 625 males and 653 females included in the sample. Of the total images, 500 patients met the inclusion criteria, with 236 (47.2%) males and 264 (52.8%) females (Figure 1).

The median age of the patients was 41 years, with an IQR of 30 to 51 years. The median age of male patients was 41 years, with an IQR of 30 to 51 years, while the median age of female patients was 40.5 years, with an IQR of 29 to 52 years. Table 1 shows the baseline characteristics of the included patients.

**Table 1:** Baseline characteristics of included patients. IQR: interquartile range

Characteristics	N = 500
Gender, n (%)	236/264
Males	236 (47.2%)
Females	264 (52.8%)
Males’ age (years), median (IQR)	41.0 (30–51)
Females’ age (years), median (IQR)	40.5 (29–52)

Presence of the third root on the right side

Our findings show that 37 (7.4%) patients had right-sided third roots. Of the male patients, 11 (4.7%) had right-sided third roots, accounting for 2.2% of the total population. On the other hand, 26 (9.9%) female patients, accounting for 5.2% of the total population, also had the third root present. However, it was absent in 225 (45%) males and 238 (47.6%) females of the total population. Overall, the third root was present in 37 patients, accounting for 7.4% of the total population. Based on our analysis, we observed a significant association between gender and the presence of the third root on the right side (p = 0.027), as shown in Table 2. The type of right-sided third root was entomolaris in 34 (91.9%) patients with right-sided third root, in males, nine (81.8%) had entomolaris, while two (18.2%) had paramolaris. While in females, 25 (96.2%) had entomolaris, and one (3.8%) female had right-sided paramolaris third root.

**Table 2:** Presence and type of right- and left-sided third root between males and females. ^#^: chi-square and Fisher’s exact tests. ^*^: percentages are out of the number of right/left-sided or bilateral third roots.

Characteristics	Males, N = 236	Females, N = 264	Total, N = 500	P-value^#^
Right-sided third root				0.027
Present, n (%)	11 (4.7%)	26 (9.9%)	37 (7.4%)	
Absent, n (%)	225 (95.3%)	238 (90.1%)	463 (92.6%)	
Type of right-sided third root^*^				0.200
Entomolaris, n (%)	9 (81.8%)	25 (96.2%)	34 (91.9%)	
Paramolaris, n (%)	2 (18.2%)	1 (3.8%)	3 (8.1%)	
Left-sided third root				0.071
Present, n (%)	4 (1.7%)	12 (4.5%)	16 (3.2%)	
Absent, n (%)	232 (98.3%)	252 (95.5%)	484 (96.8%)	
Type of left-sided third root^*^				-
Entomolaris, n (%)	4 (100%)	12 (100%)	16 (100%)	
Paramolaris, n (%)	0 (0%)	0 (0%)	0 (0%)	
Bilateral third root, n (%)				-
Present, n (%)	3 (1.3%)	10 (3.8%)	13 (2.6%)	
Absent, n (%)	233 (98.7%)	254 (96.2%)	487 (97.4%)	
Type of bilateral third root^*^				-
Entomolaris, n (%)	3 (100%)	10 (100%)	13 (100%)	
Paramolaris, n (%)	0 (0%)	0 (0%)	0 (0%)	

Presence of the third root on the left side

Of the total population, 16 (3.2%) had left-sided third roots. Among males, the left-sided third root was present in 4 (1.7%) patients, accounting for 0.8% of the entire cohort. In females, the left-sided third root was present in 12 (4.5%) patients, accounting for 2.4% of the full cohort. There was no significant association between the presence of left-sided third root and gender (p > 0.05). All left-sided third roots were entomolaris.

Bilateral third root

A total of 13 (2.6%) patients had bilateral third roots. Of the patients, three (23.1%) were males, accounting for 0.6% of the total cohort, while 10 (76.9%) females had bilateral third roots, accounting for 2% of the total cohort. However, it was absent in 233 (46.6%) males and 254 (50.8%) females of the total population. All patients with bilateral third roots had entomolaris. Based on our analysis, we observed a non-significant association between gender and the presence of bilateral appearance of the third root (p = 0.07).

**Figure 2:**
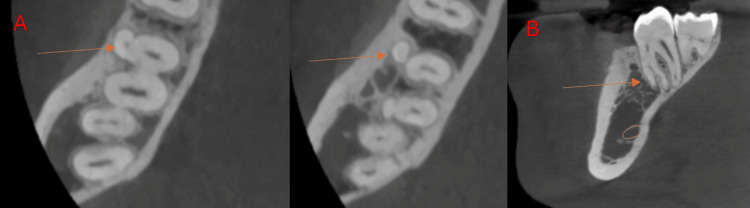
Cone-beam computed tomography images of the mandibular first molar at the right side showing radix Paramolaris (arrow). (A) Axial view. (B) Sagittal view.

**Figure 3:**
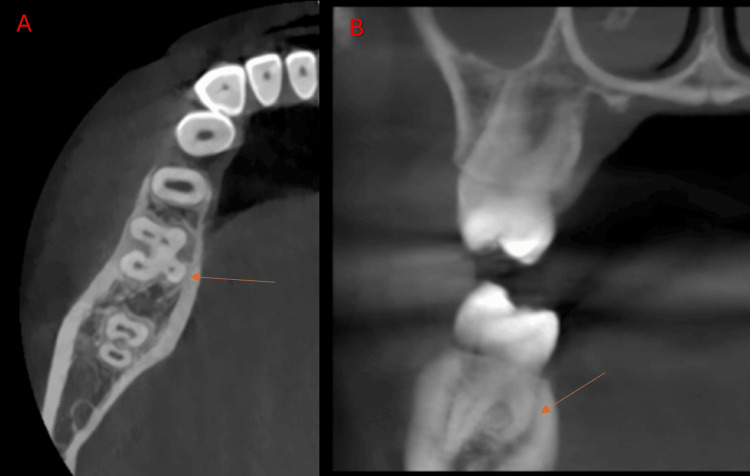
Cone-beam computed tomography images of the mandibular first molar at the right side showing radix endomolaris (arrow). (A) Axial view. (B) Coronal view.

**Figure 4:**
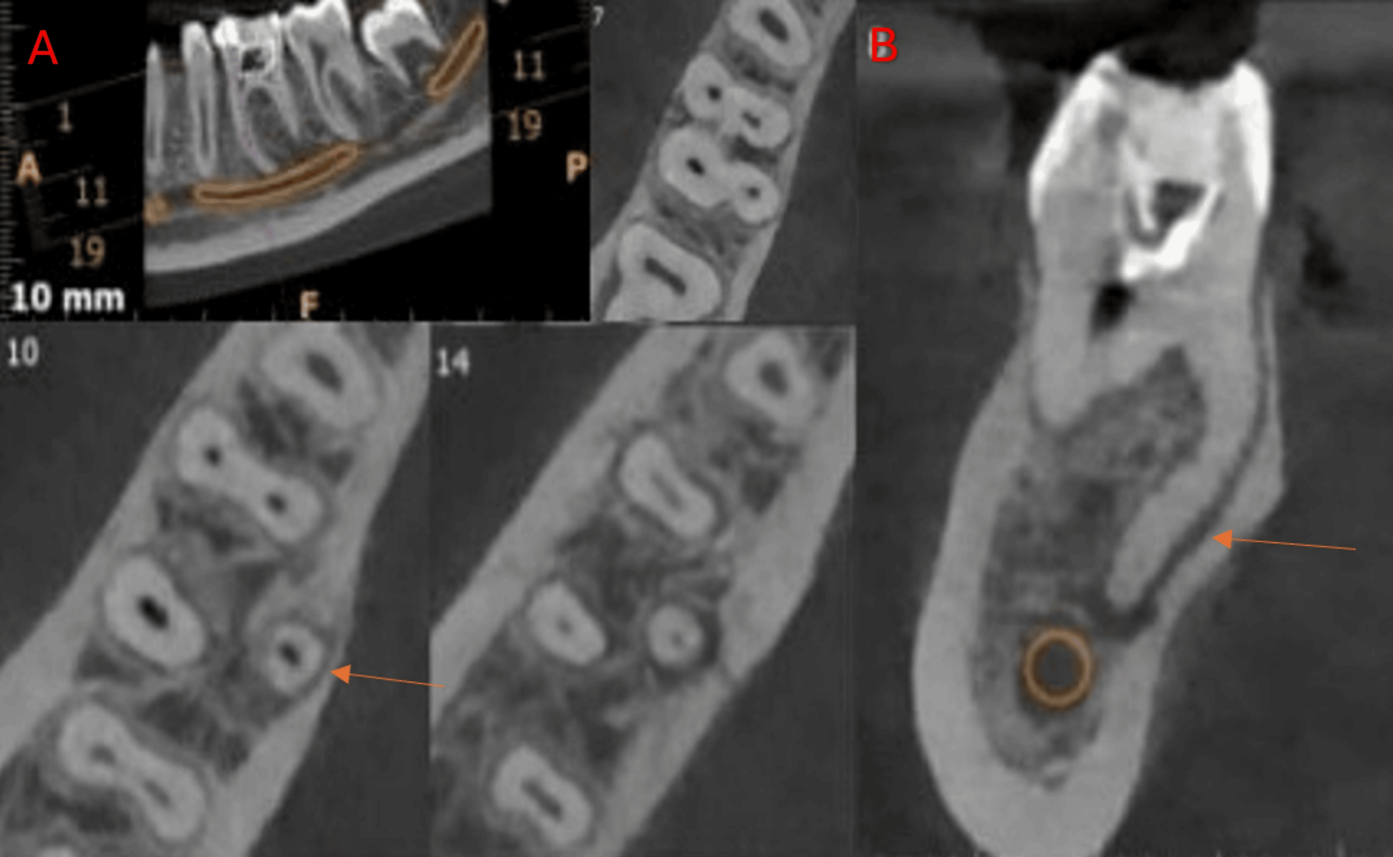
Cone-beam computed tomography images of the mandibular first molar at the left side showing radix endomolaris (arrow). (A) Axial view. (B) Coronal view.

**Figure 5:**
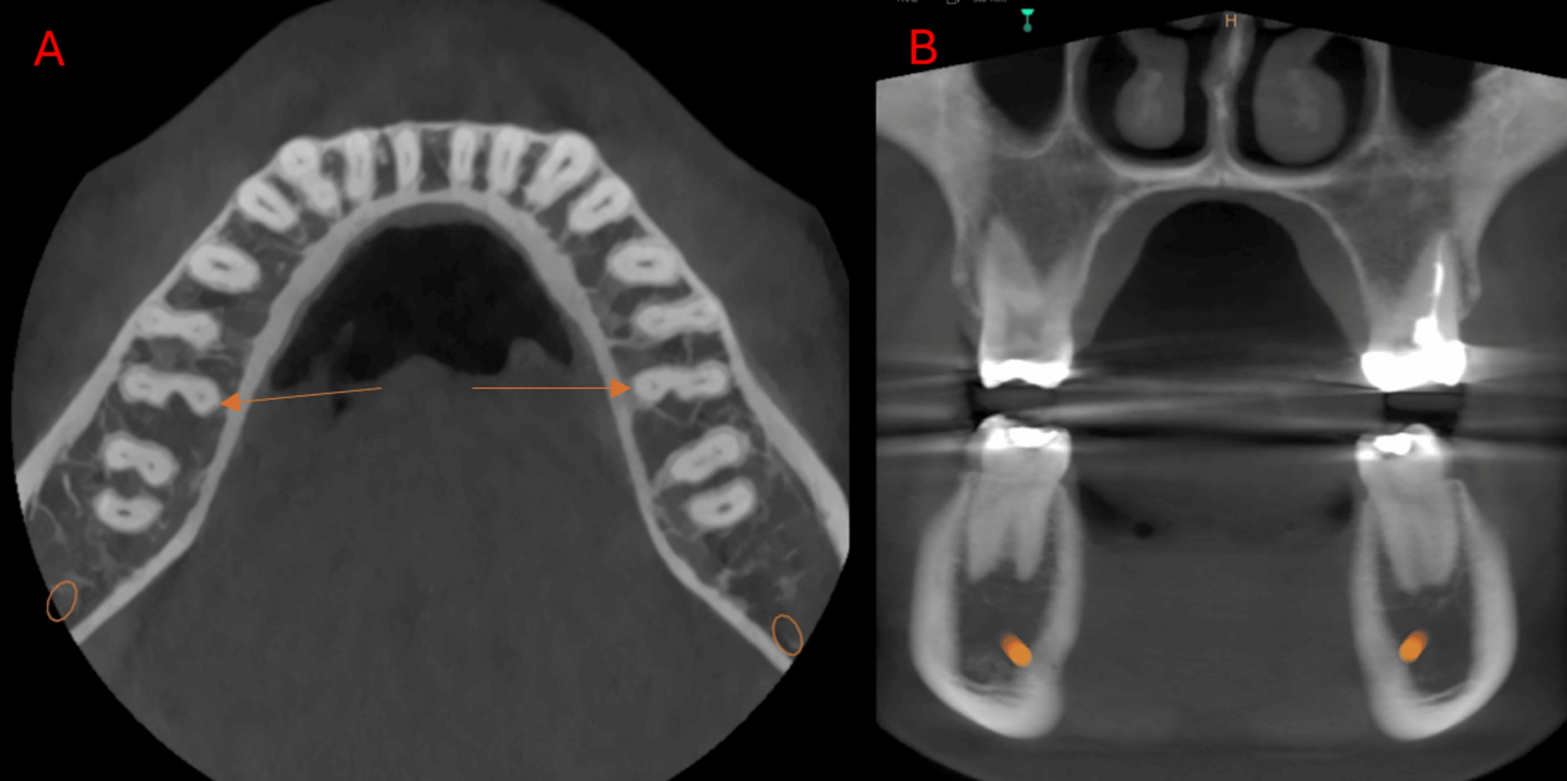
Cone-beam computed tomography images of the mandibular first molar showing the bilateral radix endomolaris (arrow). (A) Axial view. (B) Coronal view.

**Figure 6:**
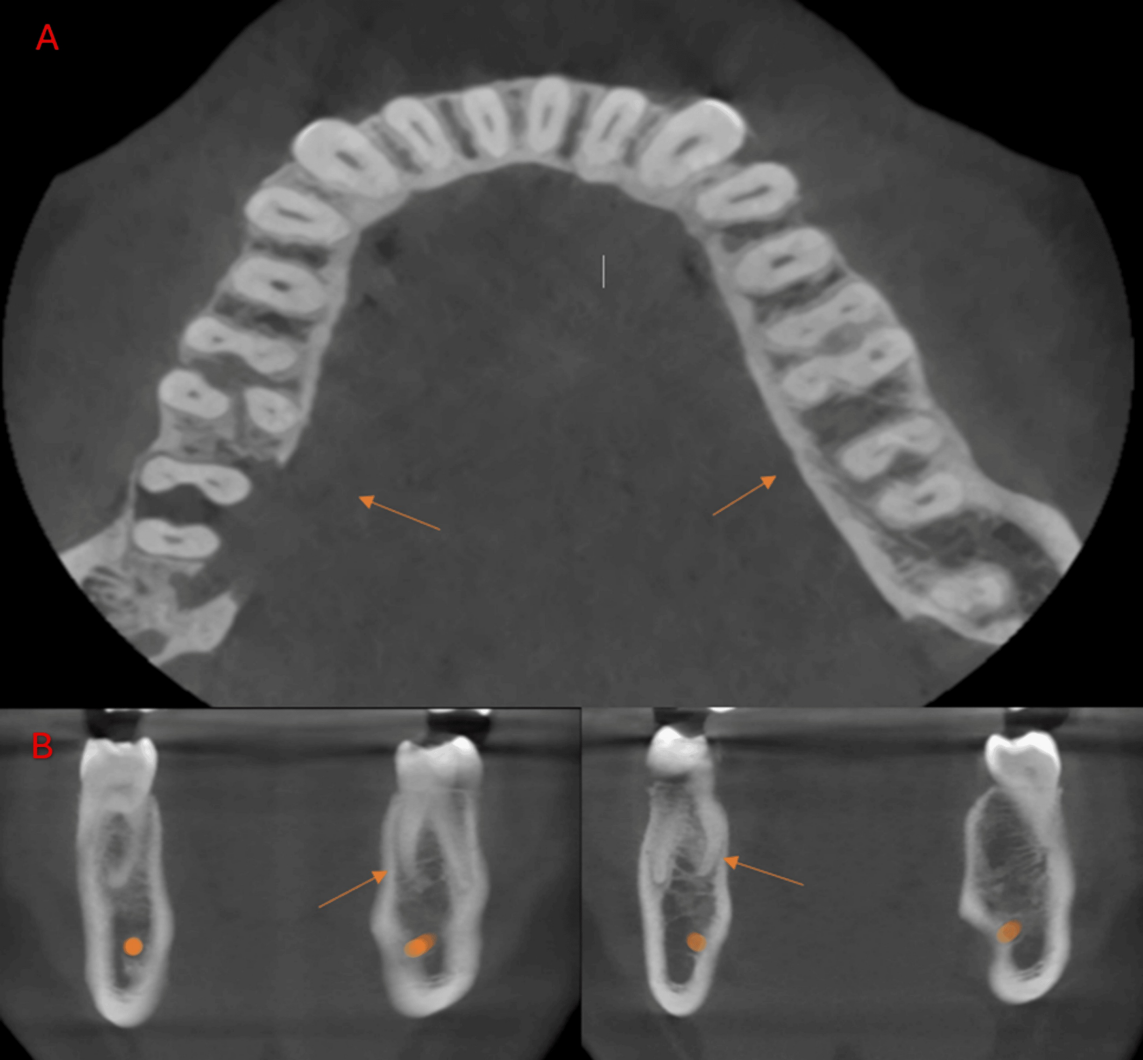
Cone-beam computed tomography images of the mandibular first molar showing the bilateral radix entomolaris (arrow). (A) Axial view. (B) Coronal view.

Figures 2-6 show some sample CBCT images of the patients included in our study.

## Discussion

Variations in root canal anatomy are among the most common factors influencing the outcome of endodontic treatment. Knowledge of this variation by clinical operators can significantly influence the outcome [8]. Therefore, familiarity with abnormal variation and its prevalence among the population is crucial. In this study, we evaluated the CBCT images of 500 patients in Jordan with fully developed bilateral mandibular first permanent molars in Jordan and compared gender disparities and the anatomical variation of the first mandibular molar.

In this study, we used CBCT images as they offer significant advantages over traditional radiography. Since its introduction into the dental field, CBCT has been widely utilized in endodontic research due to its ability to provide three-dimensional images, overcoming the limitations of conventional radiographs. CBCT has been shown to be highly accurate in identifying endodontic complexities such as extra roots, canals, and resorption [9,10]. Traditional radiography often struggled with issues such as the superimposition of roots, making it difficult to detect structures such as distolingual roots [11]. Therefore, CBCT was chosen as the imaging method in this study to ensure precise and comprehensive evaluation.

In total, 500 patients were enrolled in our study. The prevalence of the three-rooted first mandibular molar was 7.4% on the right side, 3.2% on the left side, and 2.6% bilaterally. Additional roots in mandibular permanent molars were most frequently found in races of Mongoloid origin [12]. Their incidence in first molars has been reported to lie in the range of 5.8% to 43.6% [4]. In the Arab population, however, the literature reporting the prevalence of radix entomolaris and paramolaris is scarce. The reported prevalence of radix entomolaris was 3% in the Sudanese population, 2.9% in the Saudi Arabian population, 3.1% in the Yemeni population, 3.7% in the Palestinian population, and 3.1% in the Egyptian population [13-17]. In the Jordanian population, a study by Al-Qudah et al. reported the prevalence of additional root (radix entomolaris) in the first mandibular molars to be 4% [18]. This is in line with our results, showing a prevalence rate of radix entomolaris on the right side of 6.8%, 3.2% on the left side, and 2.6% bilaterally.

In our study, a significant gender difference was found on the right-sided third mandibular root, in which females had a higher prevalence of right-sided third mandibular root compared to males (9.9% vs. 4.7%). The prevalence of right-sided radix entomolaris was higher in females compared to males (9.5% vs. 3.8%). However, there was no significant difference which is similar to the findings of other studies [14,19]. There was no significant difference regarding the position of radix entomolaris either bilateral or unilateral. This observation is consistent with the findings of earlier studies that reported a 50-69% bilateral prevalence of radix entomolaris. Controversy is present in the literature as some studies reported right-sided prevalence while few others reported left-side prevalence. A study by Karabucak et al. (2016) reported that the distolingual canal in mandibular first molars was associated with 62% of the untreated canals [20]. Furthermore, distolingual root or radix entomolaris is considered one of the most common genetic racial traits in mandibular molars [1].

Our study provides several strong points. First, we evaluated the prevalence of anatomical variation of the first mandibular molars using CBCT images instead of conventional radiography which provides a superior ability to capture a three-dimensional view compared to conventional radiography which can obscure important structural details. Second, we evaluated the anatomical variations at the right and left sides and bilaterally and reported the prevalence of additional roots separately. However, our results should be interpreted carefully due to some limitations. Conducting the study at a single center may limit the applicability and generalizability of the results to other settings or regions with different demographics or dental practices.

## Conclusions

The prevalence of additional roots in different population groups is known to exist. This study confirms a 7.4% and 3.2% prevalence of right- and left-sided radix para- and entomolaris, respectively, in mandibular first molars in a Jordanian population. Practitioners should be aware of the risks involved with missing canals during root canal treatment. Thus, proper diagnostic methods should be performed to predict the presence and locate difficult-to-find or extra root canals.
